# Editor – Human Digital Twin in Ukraine: Converging Digital Health and Digital Education for Next-Generation Telerehabilitation

**DOI:** 10.63144/ijt.2025.6729

**Published:** 2025-12-12

**Authors:** Kyrylo S. Malakhov, Dmytro V. Vakulenko

**Affiliations:** 1 MSc, Research fellow in IT, Microprocessor Technology Lab, V.M. Glushkov Institute of Cybernetics of the National Academy of Sciences of Ukraine; 2 DSc (Biological Sciences), PhD (Computer Science), Full Professor, Head of the Department of Medical Informatics, Ternopil National Medical University, Ukraine

**Keywords:** Artificial intelligence, Clinical education, Digital education, Digital health, eHealth, Human digital twin, Ukraine

## Abstract

Ukraine’s current rehabilitation and healthcare challenges have catalyzed a national push toward scalable, AI-enabled digital health solutions. This Letter reports on a joint initiative of I.Ya. Horbachevsky Ternopil National Medical University, and the V.M. Glushkov Institute of Cybernetics of the National Academy of Sciences of Ukraine to operationalize the human digital twin (HDT) concept within a tele-diagnostic (TD) & artificial intelligence (AI) platform for telerehabilitation and digital education. The Letter delineates how HDTs, understood as continuously updated virtual representations of individual patients, are coupled with AI agents to support remote patient monitoring, decision-support, and personalized rehabilitation trajectories. Beyond clinical care, these HDTs function as virtual patients for simulation-based training and in-silico experimentation, enabling students, residents, and multidisciplinary teams to rehearse diagnostic and therapeutic strategies without risk to real patients. By integrating sensor-derived data, AI-driven expert systems, and web-based learning environments, the TD+AI platform exemplifies how resource-constrained settings can effectively employ HDT technologies to converge digital health and digital education. The Letter concludes by highlighting opportunities for international collaboration to co-develop, validate, and scale such HDT-centered telerehabilitation ecosystems.

**November 24, 2025**

**Dear Ellen R. Cohn, PhD, CCC-SLP, ASHA-F and Jana Cason, DHSc, OTR/L, FNAP, FAOTA:**

Warm greetings are conveyed from Ukraine on behalf of our research teams, together with a wish to share notable advancements in human digital twin (HDT) technologies that are reshaping digital health and digital education in the Ukrainian context.

In recent years, collaborative efforts between I.Ya. Horbachevsky Ternopil National Medical University (TNMU) and the V.M. Glushkov Institute of Cybernetics of the National Academy of Sciences of Ukraine (Institute of Cybernetics) have been directed toward the development of a personalized tele-diagnostic and artificial intelligence (TD + AI) platform. Within this platform, the HDT concept is being operationalized to support telerehabilitation, remote clinical decision-support, and simulation-based medical education, with particular emphasis on multidisciplinary rehabilitation pathways and in-silico experimentation (Petrenko & Malakhov, 2025).

## Ukraine’s Digital Health Initiative and Rehabilitation Needs

Ukraine is undergoing a rapid expansion of digital health and telerehabilitation in response to extraordinary circumstances. The war has resulted in a large population of injured individuals requiring rehabilitation, many of whom cannot easily access in-person services. Consequently, there is a critical need to expand tele-diagnostic services and rehabilitation centers, and to optimize their work. Modern digital technologies, including personalized telemedicine tools, telerehabilitation platforms, and AI applications are seen as key to improving the accessibility and quality of rehabilitation services, ensuring a high level of care regardless of patients’ geographic location. This national imperative builds on earlier drivers like the COVID-19 pandemic (Velychko et al., 2021) and aligns with Ukraine’s ongoing eHealth reforms (Malakhov, 2023), which have established electronic medical / health records and telemedicine regulations.

**Figure 1:**
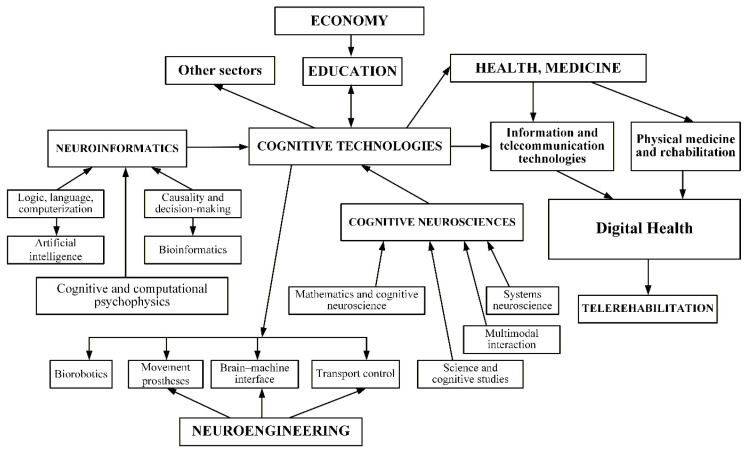
Transdisciplinary Relationships Between Cognitive Technologies, Multiple Scientific Domains, and the Ecosystems of Digital Health and Telerehabilitation

In 2025, a national R&D initiative was launched to develop a personalized TD platform powered by AI technologies, led by a collaboration between TNMU, and the Institute of Cybernetics *– “Development of a Personalized Tele-Diagnostic Platform with AI for Physicians and Patients (TD + AI)”* (Vakulenko, 2025). The goal is to integrate cutting-edge digital innovations into the healthcare system to increase the efficiency of diagnosis, and treatment, provide personalized telemedical support, and thus help reduce premature mortality. Central to this initiative is employing AI for medical data analysis (Vakulenko, Hevko, et al., 2025), enabling more accurate risk prediction, and optimized treatment planning. Equally important is a transdisciplinary approach (Malakhov et al., 2024; Palagin, 2013, 2021; Palagin et al., 2024; Petrenko & Malakhov, 2025): bridging medicine, computer science, engineering, and social sciences is considered essential for creating effective remote rehabilitation services (Vakulenko et al., 2023). By uniting expertise across these domains, the project addresses not only clinical needs but also technology integration, data governance, and user-centered design (Petrenko & Malakhov, 2024, 2025). Figure 1 schematically illustrates the network of relationships between cognitive technologies, multiple scientific domains, digital health, and telerehabilitation, thereby providing a visual representation of this transdisciplinary approach.

## AI-Based Human Digital Twin for Telerehabilitation, Education, and In-Silico Modeling

A central element of Ukraine’s digital health strategy, and of the TD+AI project in particular, is the *Human Digital Twin* concept. In engineering, a digital twin is a detailed virtual model of a physical object or process. In healthcare, an HDT is a virtual patient model that reproduces the state of an individual’s body and can be continuously updated with real data to predict changes in health status. This entails aggregating heterogeneous patient data from electronic medical / health records, sensors, imaging, and wearables into a continuously evolving representation of the individual. Such technology enables a spectrum of applications, from personalized prevention to simulating treatment trajectories on a virtual patient without risk to the real person. In practical terms, an HDT serves as a virtual patient that mirrors a person’s physiology and clinical trajectory (Vakulenko, Vakulenko, et al., 2025).

This concept is being combined with advanced AI agents that act as virtual clinicians. Contemporary large language models (LLM) and related AI systems, especially those tuned for medical tasks, are increasingly capable of engaging in natural dialogue, analyzing symptom descriptions, and suggesting preliminary diagnostic and therapeutic options. Experimental *“virtual hospital”* configurations have emerged in which AI agents assume roles analogous to doctors, nurses, and patients, simulating clinical scenarios that range from routine consultations to complex interventions. The novelty of the present approach lies in uniting HDT-based virtual patients with AI-driven virtual clinicians in a single workflow that spans digital education, teleconsultation, and clinical decision support. When AI-powered virtual clinicians interact with patient digital twins, a safe and information-rich environment is created for both training and care delivery.

In telerehabilitation, this framework augments the capabilities of human providers. Computer vision and machine learning models can monitor a patient performing exercises at home via a camera, automatically evaluating movement quality and providing immediate corrective feedback (including biofeedback (Kaverinskiy et al., 2024; Kurgaev & Malakhov, 2026)). If an exercise is executed incorrectly, the system can adjust technique through a virtual coach avatar; if adherence declines, automated reminders and motivational messages can be issued. During a live video rehabilitation session, the human rehabilitation physician may be accompanied by several AI assistants, for example virtual consultant agents specialized in physiotherapy, neurology, or cardiology. As the patient performs movements, these agents analyze biomechanical and neurological parameters in real time (such as range of motion, gait symmetry, or indicators of neuromuscular impairment) and propose optimal adjustments to the rehabilitation program (Vakulenko et al., 2024). In this configuration, the encounter effectively becomes a *virtual concilium* in which one or more human clinicians and multiple AI agents jointly review the state of the human digital twin, discuss options, and co-produce clinical and rehabilitative decisions. The patient thus benefits from the combined expertise of a multidisciplinary team, even when geographically only a single human clinician is present.

**Figure 2:**
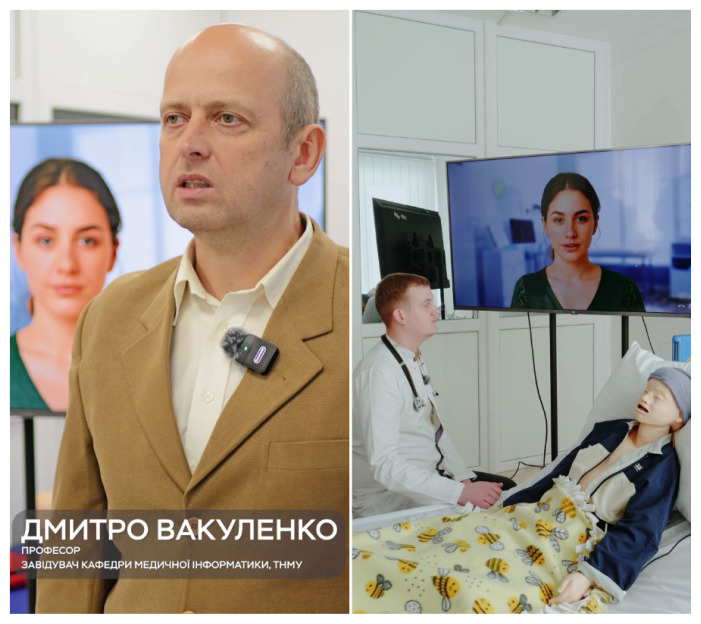
AI-driven Virtual Patient Avatar in a Real-world Digital Health and Digital Education Use Case. Left: Professor Dmytro V. Vakulenko. Right: Simulated Pediatric Respiratory Case in Which a Clinician Conducts a Teleconsultation with an AI avatar and a Pediatric Manikin. All individuals depicted in Figure 2 provided informed consent for the use of their images in this Letter.

An illustrative real-world implementation of this configuration at TNMU is presented in Figure 2. The left panel shows Professor Dmytro V. Vakulenko, Head of the Department of Medical Informatics and overall leader of the TD+AI project. The right panel depicts a simulated teleconsultation in which a clinician interacts with an AI-driven virtual avatar and a high-fidelity pediatric manikin representing a child with respiratory disease, exemplifying how virtual concilium formats with HDT and AI agents can be embedded into routine digital health workflows and simulation-based education.

The HDT concept also provides powerful tools for clinical education and in-silico research (Petrenko & Malakhov, 2025). Virtual patients enable medical students and trainees to practice diagnostic reasoning and therapeutic decision-making in realistic, dynamic simulations. Unlike static manikins or purely text-based case studies, a digital twin can incorporate real patient data and evolving pathophysiology. In cardiology training, for example, digital three-dimensional heart models derived from individual imaging datasets allow surgeons to visualize and rehearse an operation on a virtual heart prior to the real intervention. Similarly, AI-driven patient avatars can reproduce symptom patterns and disease dynamics with sufficient fidelity to support complex virtual clinical cases. This immersive simulation paradigm enhances the preparedness of young specialists while maintaining absolute safety, as any diagnostic or therapeutic error affects only the virtual patient. Furthermore, the combined HDT and AI-agent framework enables experimentation with new treatment and rehabilitation strategies entirely in silico. Different rehabilitation protocols or medication regimes can be evaluated on cohorts of patient twins to identify promising or potentially harmful trajectories before application in vivo. Such in-silico trials accelerate innovation and reduce risk in the design of telerehabilitation pathways.

To illustrate these developments, two complementary video resources are available. The first is a demonstration of a HDT and AI avatar in a real-world digital health use case at TNMU (*Human Digital Twin & AI Avatar for Digital Health*, 2025). The second is a keynote presentation delivered at the 5th National Congress of Physical and Rehabilitation Medicine “Physical and Rehabilitation Medicine in Ukraine,” (*The 5th National Congress of Physical and Rehabilitation Medicine*, 2025) which discusses the broader conceptual and architectural integration of AI agents and HDT into digital medicine (*Integration of AI Agents and HDT into Digital Medicine*, 2025). Together, these materials exemplify how human digital twin technologies and AI agents can be instantiated within digital health and digital education ecosystems to support next-generation telerehabilitation.

## Technical Implementation of the TD+AI Platform

The TD+AI platform is implemented as an integrated telehealth ecosystem that combines Internet of Medical Things (IoMT) devices, cloud services, AI-driven expert systems, and specialized user interfaces for patients, clinicians, and learners. Its architecture is designed to support continuous data flow between the physical patient and the HDT, while remaining robust in environments with constrained or unstable connectivity.

### Data Acquisition via IoMT Devices and Sensors

Patients are equipped with a set of medical devices and sensors that collect biomedical signals and functional parameters relevant to diagnosis and rehabilitation (Vakulenko & Vakulenko, 2024). These IoMT components include, for example, blood pressure monitors, ECG recorders, motion and posture assessment systems, thermal cameras, and portable devices for joint mobility monitoring. The devices communicate via Bluetooth or Wi-Fi with a patient-side gateway, typically a smartphone or tablet, which aggregates measurements and prepares them for secure transmission. This configuration enables continuous or intermittent remote monitoring of cardiovascular, neuromuscular, and musculoskeletal status, as well as basic activity and adherence indicators.

### Mobile Application, Cloud Services, and Personal Health Portals

A dedicated mobile application acts as the primary interface between the patient and the TD+AI infrastructure. It orchestrates data collection from IoMT devices, encrypts and transmits measurements to a secure cloud back end, and provides patients with a personal digital cabinet where historical and current health indicators are presented. On the server side, a cloud service receives, stores, and processes incoming data streams and synchronizes them with the corresponding HDT. This arrangement supports bi-directional communication: new measurements update the digital twin, and AI-generated recommendations or alerts are returned to the mobile application and patient portal.

### AI-driven Decision Support and Hybrid Expert Systems

At the core of the TD+AI platform is an AI-based decision-support layer that aggregates multimodal data and produces clinically relevant insights for physicians and patients. This layer combines traditional rule-based components, ontology-driven reasoning, and transformer-based language models within hybrid expert systems.

A key element is the hybrid expert system MeDeBERTaBot for decision support in physical and rehabilitation medicine (Kaverinskiy & Malakhov, 2025). MeDeBERTaBot represents the evolution of earlier systems MedLocalGPT (Malakhov, 2024), and MedRehabBot (Kaverinskiy & Malakhov, 2023). In this lineage, MedLocalGPT demonstrated local, CPU-efficient LLM deployment (*Deploying LLMs on CPU-Only Environments with Llama.Cpp Library Set: MedLocalGPT Project Case*, 2025) with retrieval-augmented generation over rehabilitation and telerehabilitation corpora, while MedRehabBot introduced ontology-guided dialogue for rehabilitation decision support. MeDeBERTaBot consolidates and extends these capabilities into a single hybrid architecture that:

- uses an ontology-inspired knowledge base (Palagin et al., 2025) and structured clinical content to constrain and ground LLM outputs;
- supports intent recognition and semantic parsing of clinician and patient queries;
- generates context-aware responses and recommendations aligned with physical and rehabilitation medicine practice;
- can be invoked as a back-end expert service by various front-end clients, including the TD+AI clinician dashboard and digital education tools.

In the TD+AI platform, the MeDeBERTaBot layer and related AI pipelines process incoming patient data, clinical documentation, and free-text queries to provide triage support, risk stratification, and suggestions for diagnostic tests, rehabilitation trajectories, and self-management instructions. Importantly, all AI outputs are presented as recommendations that remain under the supervision and responsibility of human clinicians.

### Clinician Workspace and Integration with Rehabilitation Information Systems

Healthcare professionals access the TD+AI platform through a web-based clinician workspace. This interface integrates time-series data from IoMT devices, results of AI-driven analyses, and conventional clinical documentation into a unified patient view. From this workspace, clinicians can:

- inspect longitudinal trends in physiological and functional indicators;
- review AI-generated alerts and suggested rehabilitation program modifications;
- document clinical decisions and interventions;
- orchestrate multidisciplinary rehabilitation plans involving physical therapists, physiatrists, psychologists, and other specialists.

The platform is designed to interoperate with broader medical information systems for rehabilitation, enabling linkage with electronic medical records, scheduling modules, and reimbursement-related workflows where appropriate.

### OpenWebUI-based Digital Education and HDT Control Environment

On top of the core TD+AI components, a deeply customized *OpenWebUI* (*OpenWebUI*, 2025) environment has been deployed and adapted as a specialized digital space for managing large language models and Human Digital Twins, and for supporting digital education.

A dedicated OpenWebUI-based environment has been configured for centralized management, operation, and access to large language models, both locally hosted and accessed through external providers such as OpenAI API and OpenRouter API. Within this environment, integration has been implemented with services including OpenAI, ElevenLabs, and Trulience, enabling multimodal human–machine interaction that encompasses:

- text-based conversational interfaces;
- speech recognition and text-to-speech synthesis for voice-based interaction;
- interaction with a patient avatar, representing the HDT in a visually and behaviorally realistic manner.

**Figure 3:**
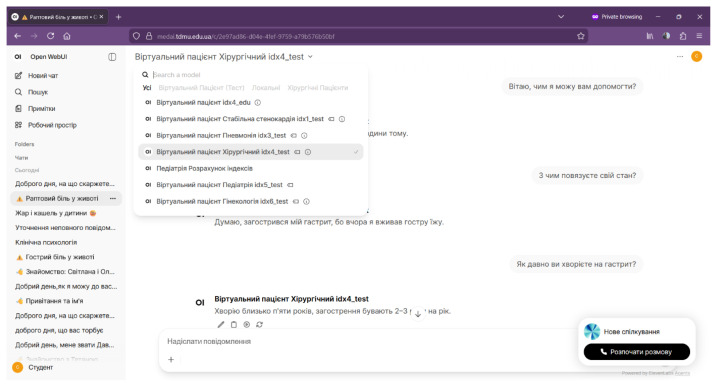
OpenWebUI-based Environment for Digital Education: Text-based Dialogue with a Virtual Patient

In routine use, this environment supports both clinical training and supervised experimentation with virtual patients, as illustrated in Figure 3. The example shows a text-based consultation scenario in which a learner interacts with a surgical virtual patient (HDT instance) in Ukrainian, selects among multiple virtual patient profiles, and conducts structured anamnesis and clinical reasoning within the OpenWebUI interface.

OpenWebUI has been substantially modified to support the HDT concept, treating HDTs as virtual patients and virtual specialists of different medical profiles, with the ability to participate in virtual concilia for clinical decision support. In such scenarios, real clinicians, virtual doctor agents, and the patient’s digital twin can be convened in a shared digital session to discuss clinical findings, explore alternative rehabilitation strategies, and document agreed decisions.

Particular emphasis has been placed on full support of local language models to ensure operational continuity in settings with limited or unstable access to external cloud services. In the current configuration, the environment includes, among others:

- gpt-oss:20B, serving as the main local HDT core for complex reasoning and longitudinal dialogue;
- MedGemma 4B and MedGemma 27B, specialized models for local analysis of medical images and multimodal content;
- Phi-4-mini-instruct, OpenBioLLM-8B, and additional open-source models for translation, summarization, and domain-specific reasoning tasks.

This OpenWebUI-based layer functions simultaneously as: a unified interaction endpoint for the TD+AI platform, and associated HDT services; a digital education platform where students, residents, and practicing clinicians can engage with virtual patients, run in-silico simulations, and interact with AI tutor agents.

Collectively, these technical components provide a resilient foundation for telerehabilitation and digital health services that can operate effectively even in resource-constrained environments and during disruptions of external cloud infrastructure.

## Future Perspectives and Collaboration Opportunities

The combined use of HDT, AI agents, and hybrid expert systems such as MeDeBERTaBot, embedded within the TD+AI platform and the customized OpenWebUI environment, delineates a coherent trajectory toward next-generation telerehabilitation and digital education. In this configuration, HDTs act as virtual patients continuously synchronized with real-world data; AI agents function as virtual clinicians capable of participating in virtual concilia with human specialists; and the OpenWebUI layer provides a unified interaction and control surface for local and cloud-based large language models, imaging models, and multimodal interfaces. Together, these components offer a reproducible, privacy-preserving, and resource-aware testbed for simulation-based training, in-silico experimentation, and clinical decision support in both routine and resource-constrained conditions.

The next stage of development necessarily involves rigorous multi-center clinical and educational evaluation. Priority directions include: prospective studies assessing the impact of HDT- and AI-supported telerehabilitation on functional outcomes, adherence, and patient-reported measures; systematic integration of the TD+AI platform with existing rehabilitation information systems and national eHealth infrastructures; and structured deployment of the OpenWebUI-based digital education environment to support competency-based training, OSCE-style assessments, and continuous professional development in physical and rehabilitation medicine. Across these activities, interoperability, transparent data governance, ethical oversight, and alignment with international regulatory frameworks for medical software and AI-based decision support will be critical.

The collaborating teams at TNMU and the Institute of Cybernetics have adopted an open-science orientation, as reflected in the public release of core software components (including MedLocalGPT, MedRehabBot, and MeDeBERTaBot) and the demonstration of HDT and AI-agent capabilities via openly accessible video keynotes and practical showcases. Building on this foundation, the teams are actively seeking international partners from telerehabilitation, digital health, AI, cognitive science, human–computer interaction, ethics, and health-policy communities. A particular goal is the co-design of joint demonstrator sites, shared HDT ontologies and reference datasets, and comparative evaluation protocols that can underpin competitive proposals to major transnational funding schemes.

In this context, the Ukrainian consortium is explicitly interested in forming consortia for submissions to United States and European funding calls, including (but not limited to) NATO innovation and research contests and relevant Horizon Europe calls in digital health, AI, and rehabilitation. Collaborative proposals in these frameworks would enable scaling and cross-national validation of HDT-centered telerehabilitation ecosystems, promote interoperability of HDT platforms and expert systems, and support the development of harmonized guidelines for safe and effective AI use in rehabilitation medicine.

By providing a forum at the intersection of telerehabilitation, digital health, and interprofessional education, the International Journal of Telerehabilitation is well positioned to catalyze such collaborations. It is anticipated that the concepts, architectures, and open tools described in this Letter will stimulate dialogue and foster partnerships aimed at maturing human digital twin- and AI-enabled infrastructures that can enhance access, quality, and personalization of rehabilitation services in Ukraine and internationally.

**Sincerely**,

**Kyrylo S. Malakhov and Dmytro V. Vakulenko**

**On behalf of the collaborating research teams of V.M. Glushkov Institute of Cybernetics of the National Academy of Sciences of Ukraine and I.Ya. Horbachevsky Ternopil National Medical University**.

## References

[b1-ijt-17-2-6729] Deploying LLMs on CPU-only Environments with llama.cpp Library Set: MedLocalGPT Project Case 2025 October 25 [Video recording]. https://youtu.be/jJnsdGrOIdg

[b2-ijt-17-2-6729] Human Digital Twin & AI Avatar for Digital Health: Virtual Patient Prototype from Ukraine 2025 November 21 [Video recording]. https://youtu.be/rT2ludR8SDE

[b3-ijt-17-2-6729] Integration of AI agents and HDT into digital medicine 2025 November 21 [Video recording]. https://youtu.be/Ze8jjnlB5Fs

[b4-ijt-17-2-6729] Kaverinskiy VV Malakhov KS 2023 *MedRehabBot* (Version 1.0.0) [Python] Knowledge-Ukraine https://github.com/knowledgeukraine/MedRehabBot

[b5-ijt-17-2-6729] Kaverinskiy VV Malakhov KS 2025 *MeDeBERTaBot* [Python] Knowledge-Ukraine https://github.com/malakhovks/MeDeBERTaBot

[b6-ijt-17-2-6729] Kaverinskiy VV Vakulenko D Vakulenko L Malakhov KS 2024 Machine Learning Analysis of Arterial Oscillograms for Depression Level Diagnosis in Cardiovascular Health Complex Systems Informatics and Modeling Quarterly 2024 40 94 110 10.7250/csimq.2024-40.04

[b7-ijt-17-2-6729] Kurgaev O Malakhov KS 2026 Next-Generation Biofeedback Rehabilitation Systems: An AI-Enabled Architecture Integrating Brain– Computer Interfaces and Knowledge-Based Control Duncan LT Advances in Health and Disease 90 90 1 34 Nova Science Publishers Inc 10.52305/YBFX7381

[b8-ijt-17-2-6729] Malakhov KS 2023 Insight into the Digital Health System of Ukraine (eHealth): Trends, Definitions, Standards, and Legislative Revisions International Journal of Telerehabilitation 15 2 1 21 10.5195/ijt.2023.6599 PMC10754247 38162941

[b9-ijt-17-2-6729] Malakhov KS 2024 Innovative Hybrid Cloud Solutions for Physical Medicine and Telerehabilitation Research International Journal of Telerehabilitation 16 1 1 19 10.5195/ijt.2024.6635 PMC11249847 39022436

[b10-ijt-17-2-6729] Malakhov KS Kotlyk SV Petrenko MG 2024 Theoretical Aspects of Transdisciplinarity in Telerehabilitation International Journal of Telerehabilitation, Special Issue: Research Status Report – Ukraine 2024 1 13 10.5195/ijt.2024.6643

[b11-ijt-17-2-6729] OpenWebUI 2025 [Computer software]. https://github.com/open-webui/open-webui

[b12-ijt-17-2-6729] Palagin OV 2013 Transdisciplinarity Problems and the Role of Informatics Cybernetics and Systems Analysis 49 5 643 651 10.1007/s10559-013-9551-y

[b13-ijt-17-2-6729] Palagin OV 2021 Information Technology Tools for Controlled Evolution Journal of Automation and Information Sciences 5 9 104 123 10.34229/1028-0979-2021-5-9

[b14-ijt-17-2-6729] Palagin OV Petrenko MG Kaverinskiy VV Malakhov KS 2025 A Method for Enhancing the Efficiency of RDF/XML-Structure Processing in the Apache Jena Semantic Web Framework Cybernetics and Systems Analysis 61 3 469 486 10.1007/s10559-025-00784-w

[b15-ijt-17-2-6729] Palagin OV Petrenko MG Malakhov KS 2024 Challenges and Role of Ontology Engineering in Creating the Knowledge Industry: A Research-Related Design Perspective Cybernetics and Systems Analysis 60 4 633 645 10.1007/s10559-024-00702-6

[b16-ijt-17-2-6729] Petrenko MG Malakhov KS 2024 Systems Science: Digitalization of Transdisciplinary Research Journal of Robotics, Networking and Artificial Life 10 4 342 352 10.57417/jrnal.10.4_342

[b17-ijt-17-2-6729] Petrenko MG Malakhov KS 2025 Methodology and Practice of Interdisciplinary Knowledge Convergence in Digital Health Information Technologies and Systems 3 3 76 96 10.15407/intechsys.2025.03.076

[b18-ijt-17-2-6729] The 5th National Congress of Physical and Rehabilitation Medicine 2025 November 14 RINMON FOUNDATION https://rimon.org.ua/rehab141125/

[b19-ijt-17-2-6729] Vakulenko DV 2025 Project registration card 0225U003958 Ukrainian Institute of Scientific and Technical Expertise and Information https://dir.ukrintei.ua/view/ok/d66d3a1c297fb98a97f8ff7966f4b4cc

[b20-ijt-17-2-6729] Vakulenko DV Hevko O Vakulenko L Smachylo I Kocherga Z 2025 Telemedicine and Psychocorrection: A New Paradigm Through Healthcare Data Processing Innovations CEUR Workshop Proceedings 4057 374 386 https://ceur-ws.org/Vol-4057/paper23.pdf

[b21-ijt-17-2-6729] Vakulenko DV Palagin OV Sergienko IV Stetsyuk PI 2024 Algorithmization and Optimization Models of Patient-Centric Rehabilitation Programs* Cybernetics and Systems Analysis 60 5 736 752 10.1007/s10559-024-00711-5

[b22-ijt-17-2-6729] Vakulenko DV Vakulenko L Gandzyuk S Gandzyuk N 2025 Criteria for Assessing Status to Form Individual Training Trajectory CEUR Workshop Proceedings 4057 287 295 https://ceur-ws.org/Vol-4057/short6.pdf

[b23-ijt-17-2-6729] Vakulenko DV Vakulenko LO 2024 Arterial Oscillography: New Capabilities of the Blood Pressure Monitor with the Oranta-AO Information System Nova Science Publishers 10.52305/XFFR7057

[b24-ijt-17-2-6729] Vakulenko DV Vakulenko L Zaspa H Lupenko S Stetsyuk P Stovba V 2023 Components of Oranta-AO software expert system for innovative application of blood pressure monitors Journal of Reliable Intelligent Environments 9 1 41 56 10.1007/s40860-022-00191-4 36157718 PMC9490687

[b25-ijt-17-2-6729] Velychko VYu Petrenko MG Semykopna TV Stryzhak O Budnyk M Vladymyrov O Gorborukov V Kaverinskiy VV Golyk V Prykhodniuk V Chaikovsky I Malakhov KS Syvak O Kurgaev O Nadutenko M Shchurov O Nikitiuk D 2021 Transdisciplinary Intelligent Infomation and Analytical System for the Rehabilitation Processes Support in a Pandemic Palagin OV first V. M. Glushkov Institute of Cybernetics; PROSVITA; ITHEA® 10.54521/ibs34

